# Effects of a facilitator-delivered, group-based school intervention to improve media literacy and body dissatisfaction among adolescents: protocol of a cluster randomized controlled trial in Colombia

**DOI:** 10.1186/s13063-026-09460-6

**Published:** 2026-01-22

**Authors:** F. E. Andres, A. M. Chamorro Coneo, T. Thornborrow, M. Mebarak Chams, E. H. Evans, L. G. Boothroyd

**Affiliations:** 1 Department of Psychology, Durham University, Durham, UK; 2 Department of Psychology, Universidad del Norte, Barranquilla, Colombia

**Keywords:** Body image intervention, Colombia, Adolescents, School-based intervention, Body dissatisfaction, Media literacy, Cluster randomized controlled trial

## Abstract

**Background:**

Body dissatisfaction is highly prevalent in Latin America, including Colombia. However, culturally adapted, evidence-based interventions are lacking, although effective programs exist in Western countries. This protocol describes a cluster randomized controlled trial (cRCT) of an intervention to improve media literacy and decrease body dissatisfaction and related outcomes among adolescents in urban and rural schools in Colombia.

**Methods:**

We will recruit 1250 adolescents in 7th–10th grade (aged 11–17 years) to participate in a two-arm, cluster-randomized, open-label, controlled superiority trial in Colombia. Participating schools will be recruited by the research team. Participants will be randomized at the school level (1:1). Randomization blocks stratified by socioeconomic status (high vs. low) and geographical area (rural vs. urban) will be used to assign schools to either: (a) a school-based, four-session group intervention delivered over 2–4 weeks by trained facilitators (*n* = 625) or (b) a waitlist control group that will receive the intervention only after follow-up (*n* = 625). Participants need to be aged 11–17 years old, attending a participating school and class in grades 7 to 10, have written parental consent, and give written assent to be eligible for participation in this study. Individuals who cannot understand spoken or written language, or who do not have parental consent to participate, will be excluded. Primary outcomes are media literacy and body dissatisfaction. Secondary outcomes include appearance comparison, thin ideal internalization, curvy ideal internalization (girls only), drive for muscularity (boys only), eating disorder symptoms, and general wellbeing. Additional exploratory outcomes include risky appearance-altering behaviours (girls only), colourism, and skin colour satisfaction. Furthermore, adverse outcomes will be recorded.

Outcome assessments will happen pre-intervention (baseline; Timepoint 1), 1-week post-intervention (immediate post-test; Timepoint 2), and 9–12 months post-intervention (follow-up; Timepoint 3).

**Discussion:**

Based on pilot data, we hypothesise that the intervention group will demonstrate increased media literacy and decreased body dissatisfaction at post-test and follow-up. We also anticipate improvements in secondary outcomes. This will be the first cluster RCT to evaluate the effect of a culturally adapted body image intervention in Colombia. This trial has been registered in the ISRCTN Registry (ISRCTN15802562, https://doi.org/10.1186/ISRCTN15802562). First enrolment was on 26th of August 2025.

**Trial registration:**

This trial has been registered in the ISRCTN Registry (ISRCTN15802562, https://doi.org/10.1186/ISRCTN15802562).

**Supplementary Information:**

The online version contains supplementary material available at 10.1186/s13063-026-09460-6.

## Administrative information

**Table:** 

Title {1}	Effects of a facilitator-delivered, group-based school intervention to improve media literacy and body dissatisfaction among adolescents: Protocol of a cluster randomised controlled trial in Colombia
Trial registration {2a and 2b}	This trial has been registered in the ISRCTN Registry (ISRCTN15802562). Registered 4th of August 2025, https://www.isrctn.com/ISRCTN15802562;10.1186/ISRCTN15802562
Protocol version {3}	Version 2 of 04/12/2025
Funding {4}	UKRI Horizon Guarantee scheme grant (EP/Z533336/1)
Author details {5a}	F.E. Andres: Department of Psychology, Durham University, Durham, United Kingdom A.M. Chamorro Coneo: Department of Psychology, Universidad del Norte, Barranquilla, Colombia T. Thornborrow: Department of Psychology, Durham University, Durham, United Kingdom M. Mebarak Chams: Department of Psychology, Universidad del Norte, Barranquilla, Colombia E. H. Evans: Department of Psychology, Durham University, Durham, United Kingdom L. G. Boothroyd: Department of Psychology, Durham University, Durham, United Kingdom
Name and contact information for the trial sponsor {5b}	L. G. Boothroyd (Principal investigator) l.g.boothroyd@durham.ac.uk
Role of sponsor {5c}	The funders played no role in the design, collection, management, analysis and interpretation of data and in writing of the report and the decision to submit the report for publication

## Introduction

### Background and rationale {6a}

Body dissatisfaction is a risk factor for eating disorders, depression, and low self-esteem [1–4]. School-based interventions to reduce body dissatisfaction and media literacy are a cost-effective way to foster resilience against appearance pressures and ultimately enhance mental health across diverse socio-economic contexts [5, 6]. However, only a few interventions have been tested in Latin American countries or rural areas so far [7]. In a cluster-randomized-controlled trial evaluating a comic-based body image intervention among Indian adolescents in semi-rural schools, participants in the intervention group reported higher body esteem (partial *η*^*2*^ = 0.01), lower eating pathology (partial *η*^*2*^ = 0.008), and reduced appearance-ideal internalization (partial *η*^*2*^ = 0.13) compared with controls. These effects were maintained at 3-month follow-up [8]. In rural Nicaragua, recent improvements in media access have increased risk for body dissatisfaction and eating disorders [9, 10]. A pilot study of a culturally adapted intervention program to promote body esteem and media literacy improved participants’ media literacy and reduced their internalization of the athletic ideal. Most participants also recalled key messages 6 weeks later, indicating good acceptability and feasibility of the program [11]. The current project will establish an evidence base and theoretical framework for body image education in Colombia, through the systematic evaluation of this intervention in Colombia. In a pilot study conducted in low socio-economic neighbourhoods of Barranquilla, Colombia, the intervention demonstrated high feasibility and acceptability among both students and facilitators. It produced substantial improvements in media literacy for both boys (partial *η*^*2*^ = 0.26) and girls (partial *η*^*2*^ = 0.27) at post-test, with these large effects sustained at 4-month follow-up. Additionally, girls showed moderate increases in body esteem (partial *η*^*2*^ = 0.06) and reductions in appearance-based social comparison (partial *η*^*2*^ = 0.07), which were maintained over the same period (manuscript in preparation).

### Objectives {7}

Evaluate whether participants randomized to receive the intervention show increased media literacy, decreased body dissatisfaction, and/or improvements in secondary outcomes at timepoint 2 and timepoint 3 compared to baseline, relative to participants randomized to the control group.

Assess effects of moderators (socioeconomic status (SES), school type, geographical location) on any observed changes in primary and secondary outcomes in response to the intervention.

### Trial design {8}

In this two-arm cluster-randomized, open-label, controlled superiority trial, we will compare changes in primary and secondary outcomes in participating adolescents randomized to receive either the intervention or a wait-list control condition in a parallel group design over the first 9–12 months of the study. The group allocation ratio is 1:1, clustered by school and stratified by geographical location (urban/rural), socioeconomic position (high/low), and funding status (state/private). Participants in the waitlist control group will receive the intervention after follow-up data collection is concluded.

## Methods: participants, interventions, and outcomes

### Study setting {9}

This project will run full assessments of the effectiveness of the program with adolescents attending 7th to 10th grade (typically 11–17-year-olds) in urban and rural Northern Colombia. Trained facilitators (paid research assistants) and students will deliver materials during the school day. Schools will be recruited through word of mouth and researcher contacts. All intervention activities are classroom-based and will be delivered to mixed-gender groups of 20–40 participants during class time. Outcome assessments will also be completed during the school day, in classrooms. Materials have been refined based on prior pilot study data with adolescents in the city of Barranquilla (*N* = 338). Quantitative assessments will use a wait-list control design, with outcomes measured at baseline, immediate post-test, and follow-up (9–12 months later). We are using translated and validated scales to assess body image, sociocultural internalization, eating attitudes, and general wellbeing. Additionally, we are in the process of validating scales measuring appearance comparison, curvy ideal internalization, appearance-altering behaviours, and colourism attitudes. All scales have undergone psychometric evaluation and linguistic adaptation to ensure cultural relevance for the Colombian context in a sample of Colombian adults (*N* = 505; *M*_age_ = 21.48, *SD* = 2.96, range 18–30). 

In Colombia, all areas are classified into six socioeconomic strata (*estratos*) based on residents’ income and living conditions. *Estrato* 1 includes the lowest-income households, whereas *Estrato* 6 comprises the highest. This stratification system is used to determine public service subsidies and utility fees: lower strata receive financial assistance for basic services such as electricity and water, while higher strata pay increased rates to support social equity [12].

### Eligibility criteria {10}

Participants must meet the following criteria to be eligible for the study:

- Aged 11–17 years
- Attending a participating school and class
- Written parental consent
- Written child assent before the start of the study
- In school grade 7 to 10 (11–17-year-olds)
- Urban cluster: school is in an urban area, as classified by [13]
- Rural cluster: school is in a rural area [13]
- High socioeconomic status: schools located in areas defined as stratum (*estrato*) 4, 5, or 6
- Low socioeconomic status: schools located in areas defined as stratum (*estrato*) 1, 2, or 3.

If the participants meet any of the following criteria, they will not be eligible for the study:

- Aged ≤ 11 or ≥ 17 years
- Do not have written parental consent
- Participants with mental health conditions (e.g. active eating disorders) are not excluded from the study; however, parents are explicitly advised to think carefully about whether consenting to their child’s participation in the study is in their best interests in this situation and not to give consent if they have any concerns.

### Who will take informed consent? {26a}

Schools in Northern Colombia will be contacted by the research team and invited to participate in the study. Following approval from school principals, written study information will be distributed to parents. Parents will be required to provide written informed consent for their adolescent children to participate by returning a signed consent form to the team via the school or by completing a secure online form (hosted on Qualtrics). Adolescents (11–17-year-olds) will receive both written and verbal information about the study before filling out baseline questionnaires and will be asked to provide informed assent to participate. Only participants with valid parental consent and their own assent will be included in this study. Parental consent and adolescent assent will be collected by a member of the research team.

### Additional consent provisions for collection and use of participant data and biological specimens {26b}

Not applicable. This study is limited to self-report questionnaire data and does not involve the collection of biological specimens.

## Interventions

### Explanation for the choice of comparators {6b}

Trained facilitators (psychologists) will deliver the intervention, while participants in the control group will be assigned to a waitlist. All facilitators will undergo comprehensive training in intervention delivery. The waitlist control group will receive the same intervention from the same facilitators following the completion of follow-up assessments.

The waitlist control design was selected to allow for a comparison of intervention effects against “business as usual” for adolescents in Colombian schools, while ensuring ethical access to the program for all participants eventually. The use of a waitlist also facilitates the evaluation of long-term effects (via follow-up) before exposing the control group to the intervention. In the unlikely event that unanticipated and severe mental health outcomes emerge during implementation, we will re-evaluate the appropriateness of delivering the intervention to control groups, prioritizing the psychological well-being and ethical safeguarding of all participants. 

### Intervention description {11a}

The intervention, called “Soy Como Soy”, is a four-session, school-based intervention designed to promote positive body image (thus decreasing body dissatisfaction) and increase media literacy among adolescents in Colombia. Every session lasts 45 mins and is intended for mixed-gender classroom groups of children aged 11–17 years in their respective class age groups (20–40 participants).

The program targets the reduction of appearance-related pressures by teaching participants to critically analyse media messages, resist unrealistic appearance ideals, and support themselves and others in valuing non-appearance-related qualities. Each session incorporates psychoeducation, interactive discussions, and self-reflection exercises. The program is aligned with evidence-based change techniques for body image interventions [14] and has been pilot-tested in both Nicaragua [11] and Colombia (manuscript in preparation).

All sessions will be administered within a timeframe of 2 to 4 weeks, with either one or two sessions per week, depending on the school’s preference and availability. All sessions will be delivered during usual school hours and in a classroom setting. The facilitators will provide all materials necessary for the sessions (e.g. comic-based storyboards, paper, whiteboard markers).

### Criteria for discontinuing or modifying allocated interventions {11b}

At an early stage, intervention participation of a school can be discontinued if the school principal or staff are unresponsive or uncooperative. If only a small number of parents gave written informed consent in any given class (i.e., less than 30%) and enough consent forms cannot be obtained after a reminder, the research team can decide to switch to a different class. Recruitment will continue until the planned sample size is achieved.

Participants can leave the classroom during data collection and intervention sessions at any time without giving a reason. Participants’ participation can also be ended by facilitators if the participant is uncooperative and disruptive to the delivery of the sessions. Data that have been collected up to that moment will be included in the analysis.

A severe adverse event would be defined as an instance of significant and prolonged mental health crisis in an intervention group participant directly linked to their participation in the intervention. Facilitators will be trained to closely observe participants for signs of any distress and to immediately offer support and remove the participant from the intervention session. Facilitators will be instructed to immediately report any event that may approach the threshold for a severe adverse event to the study principal investigator (PI), in writing and, where possible, by telephone in addition. The study will be terminated prematurely if multiple adverse events occur or if the research team determines that the high ethical standards of the intervention can no longer be maintained. In case of the premature ending of the study, all participants and their parents will be informed through letters or emails. All data up to that moment will be included in the analysis. No adverse events occurred during the pilot trial of the intervention.

### Strategies to improve adherence to interventions {11c}

Adherence to the intervention will be actively monitored and promoted by both the facilitators and the head teachers, who will encourage participants to attend sessions, engage with the intervention materials, and remind them about homework. Facilitators will complete documentation to track attendance and participation throughout the program. The waitlist control group will not be required to follow any specific protocol.

### Relevant concomitant care permitted or prohibited during the trial {11d}

There are no permitted or prohibited forms of concomitant care during the study.

### Provisions for post-trial care {30}

The research team will keep in contact with the schools, even after follow-up data collection. Team member Dr. Moises Mebarak is a qualified clinical psychologist and will provide clinical referrals for any participants identified as needing follow-up assessment. No compensation will be provided to participants who experience harm because of trial participation.

### Outcomes {12}

All outcomes are self-report questionnaires and differences in scores will be compared from baseline (Timepoint 1), immediate post-test (Timepoint 2), and follow-up (Timepoint 3), as well as between intervention and waitlist control groups. All questionnaires will be administered at all three timepoints.

### Primary outcome measures

- *Media literacy* will be assessed using the *Perceived Reality Scale–Social Media (PRS-SM)*. This questionnaire is answered on a 5-point Likert scale from *strongly disagree* (1) to *strongly agree* (5). Higher average scores indicate lower media literacy (range 1–5). The scale contains 5 items and was originally developed to focus on TV [15] and later adapted for social media by the research team. This version was validated in Spanish with Colombian adults and showed good reliability, ω = 0.84 (Boothroyd et al., under review).
- *Body dissatisfaction* will be assessed using the *Body Esteem Scale for Adolescents and Adults (BESAA)*. This questionnaire is answered on a 5-point Likert scale from *never* (1) to *always* (5). Higher average scores indicate lower body dissatisfaction (range 1–5). This scale was originally validated in Canadian adults and contained 23 items [16]. The Latin American Spanish version was validated in young adults from Colombia, contains 18 items, and achieved excellent reliability, ω = 0.92 [17].

### Secondary outcomes

- *Comparison attitudes* will be assessed using the *Multidimensional Physical Appearance Comparison Scale (M-PACS)*. This questionnaire is answered on a 5-point Likert scale from *never* (1) to *almost always* (5). Higher average scores indicate more frequent appearance comparisons (range 1–5). This new version is based on the *Physical Appearance Comparison Scale* [18]. Validation of both the English and Spanish versions is underway. The 14-item scale showed a two-factor structure with good psychometric properties and excellent reliability (ω = 0.91) in a sample of young Colombian adults (manuscript in preparation).
- *Thin ideal internalization* will be assessed using the thin internalization subscale of the *Sociocultural Attitudes Towards Appearance Questionnaire-4 (SATAQ-4)*. This questionnaire is answered on a 5-point Likert scale from *strongly disagree* (1) to *strongly agree* (5). Higher average scores indicate higher thin ideal internalization (range 1-5). The questionnaire contains 4 items [19]. The Spanish version was validated in Colombian adults [20] and yielded good reliability (Cronbach’s α = 0.87).
- *Girls’ Curvy* *ideal internalization* will be assessed using the *Curvy Ideal Internalization scale* (CII). This questionnaire is answered on a 5-point Likert scale from *strongly disagree* (1) to *strongly agree* (5). Higher average scores indicate higher curvy ideal internalization (range 1–5). The original version was validated in the USA and contained 11 items [21]. The Latin American Spanish version was validated in Colombian women (*N* = 505, M_age_ = 21.48, SD = 2.96), contains 9 items, and achieved excellent reliability, ω = 0.92 (Andres et al., manuscript under review).
- *Boys’ drive for muscularity* will be assessed using the subscale *Muscularity-Oriented Body Image* (MBI) of the *Drive for Muscularity Scale* (DMS). This questionnaire is answered on a 6-point Likert scale from *never* (1) to *always* (6). Higher average scores indicate a higher drive for muscularity (range 1–6). The subscale contains 7 items [22]. The Spanish version was validated in adolescent boys from Spain and yielded excellent reliability (Cronbach’s *α* =.92) [23].
- *Eating disorder symptoms* will be assessed using the short version of the *Eating Disorder Examination Questionnaire* (*EDE-QS*), which assesses the frequency of eating disorder symptoms over the last week and contains 12 items [24]. This questionnaire is answered on a 7-point Likert scale from *0 days* (0) to *every day (6)* [7]. This version reached excellent reliability in British adults (Cronbach’s *α* =.91). We used the Spanish translation validated in adolescents from Spain [25].
- *General wellbeing* will be assessed using the *WHO-5 Wellbeing Questionnaire for Adolescents*, which contains 5 items [26, 27]. The questionnaire is answered on a 5-point Likert scale from *never* (1) to *all the time* (5). Higher average scores indicate higher general wellbeing (range 1–5). The Spanish version was validated in Colombian adolescents and yielded acceptable reliability, *ω* =.68 [28].

### Exploratory outcomes

- We will assess *attitudes toward and interest in risky appearance-altering behaviours* in girls using the *Risky Appearance-Altering Behaviours Inventory* (RAABI). This inventory is answered on a 5-point Likert scale from *I would never want to do this* (1) to *I have done this* (5). Higher average scores indicate higher interest and more positive attitudes towards risky appearance-altering behaviours (range 1–5). This scale contains 12 items and was developed by the research team. Validation of the English and Spanish versions of the inventory is underway. The scale showed good reliability in Colombian women, *ω* =.86 (Andres et al., manuscript under review).
- *Colourism attitudes* will be assessed using two subscales of the Colourism Scale: self-concept and attractiveness. This questionnaire is answered on a 5-point Likert scale from *strongly disagree* (1) to *strongly agree* (5). Higher average scores reflect a greater perceived importance of skin colour (range 1–5). The questionnaire includes 8 items [29]. The scale was translated by the research team, and preliminary results showed good reliability in Colombian adults, *ω* =.85.
- We will assess *skin colour satisfaction* using one item: “How satisfied are you with your skin colour?” answered on a Likert scale from 1 (*not at all satisfied*) to 5 (*very satisfied*).

### Participant timeline {13}

#### Sample size {14}

**Fig. 1:**
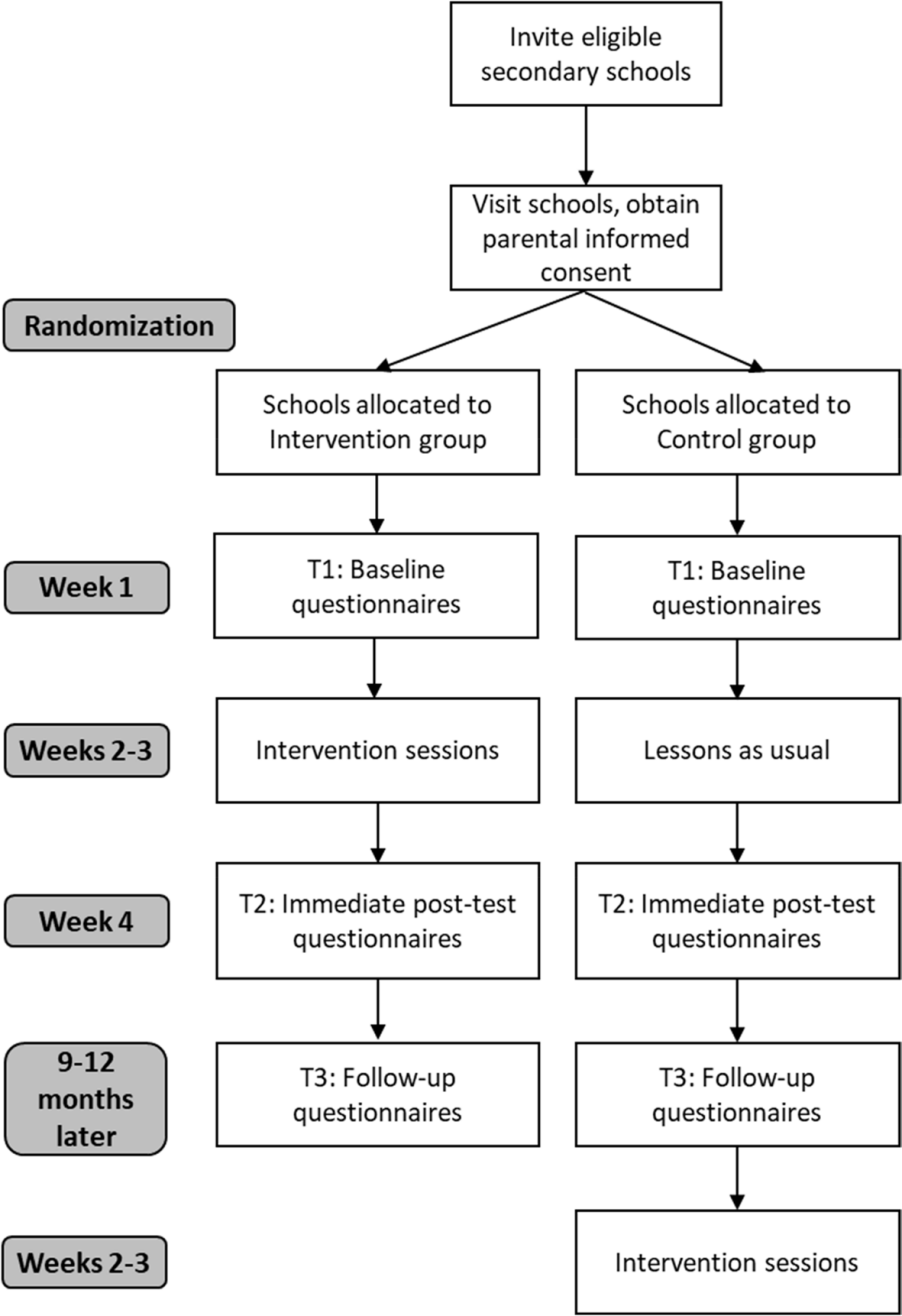
CONSORT diagram

The trial aims to detect small between-group effects of our outcomes. The pilot trial in Colombia (*N* = 338) showed that the smallest effect size was approximately *d* = 0.3 (body dissatisfaction in boys); we aimed for a slightly lower effect size of *d* = 0.25. For the primary sample-size calculation, we assumed a two-sided significance level of *α* = 0.05, 80% power (1 − β = 0.80), and equal allocation to two arms (intervention vs. control), yielding 251 participants per arm. Because this trial uses a cluster-randomized design, sample size calculations incorporated clustering effects, including ICC (intraclass correlation coefficient) and DE (design effect), which determines how much the sample size needs to be inflated compared to an individually randomized trial). Based on the pilot trial in Colombia, we assumed an average group size of *m* = 25 and an ICC of 0.046, which requires 529 participants per arm. We allowed for 10% attrition, yielding a sample size of 611 participants and 1250 participants in total (see Table 1 and Fig. 1).

**Table 1:**
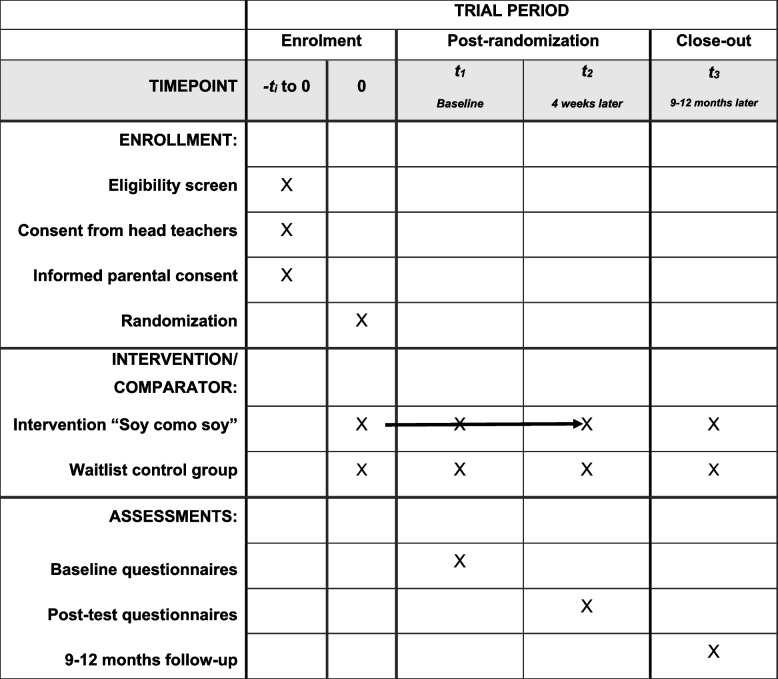
Participant timeline: schedule of enrolment, interventions, and assessments

### Recruitment {15}

Strong partnerships with schools were built through ongoing engagement activities between Universidad del Norte and local schools, beginning in 2022, which helped foster sustained interest and collaboration. Local organizations, such as Univoluntario, are involved in the project and have good connections with schools. All schools are approached via email, followed up with personal visits to the schools to present the project and receive approval from the school director. Parental consent forms will be collected during and after parent information events before the start of data collection.

Intervention sessions will be conducted during school hours, to maximize participant engagement and availability. Participants will receive little gifts (e.g. keyrings, wristbands) and snacks; the schools will receive books to thank them for their time and effort.

## Assignment of interventions: allocation

### Sequence generation {16a}

Random blocks will be used to assign participating schools according to their SES (high vs. low) and geographical area (rural vs. urban), resulting in three different blocks: rural/low SES; urban/high SES; urban/low SES. In each block, schools will be randomly assigned to either the intervention or treatment group. The allocation sequence will be determined using computer-generated random numbers from Researchrandomizer.org.

### Concealment mechanism {16b}

Allocation is not concealed and will be revealed to both participants and the research team after randomization.

### Implementation {16c}

An allocation sequence will be generated by the PI; they will assign schools to the intervention and control groups without knowing the name and exact location of the school. The study group will then be revealed to the research team and participating schools.

## Assignment of interventions: blinding

### Who will be blinded {17a}

Participants, researchers, and facilitators will not be blinded, as it is impossible for them not to realize who received the intervention and who did not. Parental consent will be obtained prior to randomization. However, schools must be informed of the intervention schedule in advance to facilitate planning, allocate time for the sessions, and arrange travel. Participants will complete the questionnaires without prior knowledge of the upcoming intervention to minimize potential bias; they will be informed afterward regarding the timing and nature of the next activity.

### Procedure for unblinding if needed {17b}

We will not blind anyone involved in this study; the unblinding procedure is therefore not needed.

## Data collection and management

### Plans for assessment and collection of outcomes {18a}

Outcome measures are self-reported questionnaires and will be answered at baseline (Timepoint 1), immediate post-test (Timepoint 2), and follow-up (Timepoint 3).

Questionnaires are described in detail in Sect. 12.

To ensure data quality, 2 control items are included in the questionnaire (e.g., “mark number 3”). Data from participants who failed both attention checks will be excluded. Research assistants are present during completion of the questionnaires to answer questions and ensure participants’ understanding of the items. Questionnaire data is then inputted into an Excel sheet by the research assistants.

All sessions are video and/or audio recorded, and 10% of sessions are assessed for facilitator’s adherence to the script by trained assessors. The same assessors showed strong interrater reliability during the pilot study in Colombia (manuscript in preparation).

### Plans to promote participant retention and complete follow-up {18b}

The research team is fostering strong, collaborative relationships with participating schools and will provide regular updates on the project’s progress. Schools will be invited to workshops and research presentations to maintain their engagement and interest throughout the study.

All sessions are co-designed by local researchers and young adults to ensure cultural relevance and youth-centred approaches. Activities have been pilot tested and received positive feedback from both adolescent participants and facilitators. To express appreciation and encourage continued participation, participants will receive small gifts and snacks after each session. When possible, alternative catch-up sessions will be arranged within a few days for any participants who are absent on the day of data collection.

Participants who choose to withdraw from the study will be invited to share their reasons, if they feel comfortable doing so. They will also be offered additional resources or referrals to appropriate specialists, as needed.

### Data management {19}

Informed consent will be obtained from parents or guardians, and assent will be obtained from adolescent participants. Self-report questionnaires will be completed on paper. All physical documents will be securely stored in a locked office accessible only to the research team.

Questionnaire responses will be entered into Excel files stored on the secure OneDrive accounts of Universidad del Norte and backed up on Durham University’s servers as soon as possible. To ensure transparency and reproducibility, all data processing and analysis steps will be documented in RStudio. The analysis scripts and anonymized datasets will be made publicly available via the Open Science Framework (OSF).

### Confidentiality {27}

Data will be stored using unique identification codes to link participants’ pre-, post-intervention, and follow-up responses. The file containing the key to these codes will be stored separately from the anonymized research data. Only the research team will have access to personally identifiable information. All analyses and publications will be based solely on anonymized data to ensure participant confidentiality.

### Plans for collection, laboratory evaluation, and storage of biological specimens for genetic or molecular analysis in this trial/future use {33}

Not applicable, we do not collect any biological specimens.

## Statistical methods

### Statistical methods for primary and secondary outcomes {20a}

Data will be analysed using RStudio [30]. First, data will be assessed for completely missing at random using Little’s test (MCAR) [31]. If data is missing at random (MAR), we will employ linear mixed-effects models to examine the effects of the intervention, using the Maximum Likelihood approach. If data is not missing at random, we will employ Multiple Imputation. The number of imputations will be decided based on m≈ %missing (as recommended by [32]). Furthermore, we will run MNAR sensitivity analyses (pattern-mixture/delta) [33].

Each outcome will be analysed using a linear mixed-effects model including the baseline measure of the outcome, gender, and the interaction between gender (boys vs. girls) and group (intervention vs. control) as fixed effects, along with timepoint (baseline, immediate post-test, follow-up). Random intercepts will be included for participant (to account for repeated measures) and nested clustering of classes within schools to reflect the cluster-randomized design. Analyses will be conducted separately for each outcome variable; primary outcomes will be used for confirmatory inference, while secondary and exploratory outcomes will be analysed separately with appropriate multiple-comparison adjustment as required. To estimate effect sizes, we will calculate partial eta squared. Following Cohen’s [34] guidelines, values of 0.01, 0.06, and 0.14 will be interpreted as small, medium, and large effects, respectively [34].

Subgroup analyses will be conducted based on baseline scores for each outcome variable, comparing participants with high versus low initial levels (categorized using median splits of baseline scores). In addition, moderator analyses will include interactions between each moderator and group to examine the potential influence of gender, socioeconomic status (high vs. low), location (urban vs. rural), and school type (state vs. private) on intervention effects.

### Interim analyses {21b}

An interim data analysis will be conducted once all immediate post-test data (Timepoint 2) have been collected. Final analyses will be performed after the completion of data collection at follow-up (Timepoint 3). This interim analysis will ensure data quality and participant safety before the follow-up phase. The intervention will be stopped if a significant increase in clinically concerning eating disorder symptoms or other adverse effects is detected.

Interim results will be shared internally with the research team, advisors, and relevant stakeholders, and may also be presented at academic conferences. These findings will not be published until the follow-up phase is complete; however, we may conduct and share secondary analyses of baseline variables prior to final publication.

### Methods for additional analyses (e.g. subgroup analyses) {20b}

Subgroup analyses will be conducted based on baseline scores for each outcome variable, comparing participants with high versus low initial levels. In addition, moderator analyses will examine the potential influence of socioeconomic status (high vs. low), geographical area (urban vs. rural), and school type (state vs. private) on intervention effects.

### Methods in analysis to handle protocol non-adherence and any statistical methods to handle missing data {20c}

Little’s MCAR test [31] will be conducted to assess whether data are missing completely at random (MCAR). Participants who have more than 40% missing item responses on a given questionnaire will be excluded from analyses involving that specific measure but will remain included in other analyses where sufficient data are available.

If data are not missing at random, we will employ Multiple Imputation. The number of imputations will be decided based on m≈ %missing responses to items (as recommended by 32). Furthermore, we will run MNAR sensitivity analyses (pattern-mixture/delta) [33].

### Plans to give access to the full protocol, participant level–data, and statistical code {31c}

Anonymized data and analytic code will be shared on the Open Science Framework (OSF).

## Oversight and monitoring

### Composition of the coordinating centre and trial steering committee {5d}

This study is being conducted across multiple secondary schools in Colombia, through a collaborative partnership between Universidad del Norte (Barranquilla, Colombia) and Durham University (UK). The project is jointly designed and implemented by both institutions. Day-to-day support is provided by the principal investigator and the UK research team, who oversee the overall trial management.

Data management is a shared responsibility between the Colombian and UK teams, with data securely stored on both institutions’ servers. The study coordinator, Dr. Ana Maria Chamorro, leads the organization of data collection, school visits, data entry, and secure data storage.

The research team holds weekly meetings in various subgroups to coordinate ongoing activities and convenes monthly as a full team to review trial progress. Communication is maintained continuously through WhatsApp and email.

An international advisory group, composed of experts in the field, meets twice a year to provide strategic guidance and oversight. The advisory group is independent of the study. Several members of the advisory group have extensive statistical knowledge.

### Composition of the data monitoring committee, its role and reporting structure {21a}

No data monitoring committee (DMC) has been appointed for this study. Since this is not a blinded study in terms of whether a participant has been allocated to the intervention vs. control condition, or in terms of outcome assessments, no DMC is required to protect the blinding of researchers and participants.

### Adverse event reporting and harms {22}

Adverse events will be reported to the principal investigator (PI), who will promptly notify the local ethics committee and the advisory group. Any necessary protocol adjustments will be communicated to the entire research team.

In cases where an adverse event requires clinical intervention—such as if a child is triggered by discussions related to an unknown eating disorder—Dr. Moises Mebarak, a clinical psychologist on the local project team, will refer the child to appropriate clinical services.

### Frequency and plans for auditing trial conduct {23}

No auditing trial has been planned for this study, as it is not a common procedure in psychological body image interventions.

### Plans for communicating important protocol amendments to relevant parties (e.g. trial participants, ethical committees) {25}

All substantial amendments will be discussed within the research team and communicated to the relevant ethics committees and the advisory group. If amendments affect participants, they will be informed of the changes, and additional consent will be obtained when necessary. Trial registries will be updated accordingly to reflect these amendments. Funders will receive a final report; no interim updates or data will be shared with them prior to project completion.

### Dissemination plans {31a}

The results of this trial will be published in international peer-reviewed journals and presented at international conferences, regardless of the study outcomes. Participating schools will also receive summary reports detailing the findings.

## Discussion

Parental consent and written assent from adolescents will be obtained prior to collecting any data before and after the intervention activities. If parental consent cannot be secured, no outcome data will be collected from those adolescents. It will be the school’s decision whether these adolescents participate in the intervention activities or engage in alternative activities during that time.

In 2024, Colombia enacted a law (Ley 2383 de 2024) mandating the integration of socio-emotional education across the national school system, recognizing the importance of mental health initiatives in fostering children’s and adolescents’ socio-emotional development. The legislation requires structured activities to strengthen socio-emotional skills and promote mental and physical wellbeing [35]. This intervention aligns closely with the new legislation, making the present RCT a timely and valuable contribution to evaluating the effectiveness of a program designed to enhance media literacy and body esteem, thereby fostering resilience among young people.

## Trial status

Recruitment has started on the 13th of August 2025. The current protocol is version 2 of the 4th of December 2025. Recruitment is estimated to be completed by August 2027.

## Supplementary Information

Additional file 1. Supplementary Materials S1-list of questionnaires

## Data Availability

Analytical code and anonymized data will be made available on the Open Science Framework (OSF).

## Abbreviations

SES: Socioeconomic status
ED: Eating disorder
OSF: Open Science Framework
BD: Body dissatisfaction
PI: Principal investigator

## References

[1] Bornioli A, Lewis-Smith H, Slater A, Bray I. Body dissatisfaction predicts the onset of depression among adolescent females and males: a prospective study. J Epidemiol Community Health. 2021Apr 1;75(4):343–8. 10.1136/jech-2019-213033 33288655

[2] Ferreiro F, Seoane G, Senra C. Toward understanding the role of body dissatisfaction in the gender differences in depressive symptoms and disordered eating: a longitudinal study during adolescence. J Adolesc. 2014Jan 1;37(1):73–84. 24331307 10.1016/j.adolescence.2013.10.013

[3] Neumark-Sztainer D, Paxton SJ, Hannan PJ, Haines J, Story M. Does body satisfaction matter? Five-year longitudinal associations between body satisfaction and health behaviors in adolescent females and males. J Adolesc Health. 2006Aug 1;39(2):244–51. 16857537 10.1016/j.jadohealth.2005.12.001

[4] Wang Y, Lynne SD, Witherspoon D, Black MM. Longitudinal bidirectional relations between body dissatisfaction and depressive symptoms among Black adolescents: a cross-lagged panel analysis. PLoS One. 2020J;15(1):e0228585. 31999799 10.1371/journal.pone.0228585 PMC6992219

[5] Guest E, Zucchelli F, Costa B, Bhatia R, Halliwell E, Harcourt D. A systematic review of interventions aiming to promote positive body image in children and adolescents. Body Image. 2022;1(42):58–74. 10.1016/j.bodyim.2022.04.009 35679652

[6] Kusina JR, Exline JulieJ J. Beyond body image: a systematic review of classroom-based interventions targeting body image of adolescents. Adolesc Res Rev. 2019;4(3):293–311.

[7] Dunker KLL, Carvalho PHBde, Amaral ACS. Eating disorders prevention programs in Latin American countries: a systematic review. Int J Eat Disord. 2023. 10.1002/eat.23916. 36789735 10.1002/eat.23916

[8] Lewis-Smith H, Ahuja L, Hasan F, Gentili C, White P, Diedrichs PC. A comic-based body image intervention for adolescents in semi-rural Indian schools: a randomised controlled trial. Int J Clin Health Psychol. 2025Jan 1;25(1):100546. 39911164 10.1016/j.ijchp.2025.100546 PMC11795790

[9] Boothroyd LG, Jucker JL, Thornborrow T, Barton RA, Burt DM, Evans EH, et al. Television consumption drives perceptions of female body attractiveness in a population undergoing technological transition. J Pers Soc Psychol. 2020;119(4):839–60. 31854999 10.1037/pspi0000224

[10] Thornborrow T, Evans EH, Tovee MJ, Boothroyd LG. Sociocultural drivers of body image and eating disorder risk in rural Nicaraguan women. J Eat Disord. 2022;10(1):1–16. 36068623 10.1186/s40337-022-00656-0 PMC9450464

[11] Andres FE, Thornborrow T, Bowie WN, Taylor Hebbert V, Allen Moses J, Gonzalez M, et al. Pilot trial assessing acceptability, feasibility, and preliminary effects of a body image intervention for adolescents in rural Nicaragua. Body Image. 2025Dec;1(55):101970. 10.1016/j.bodyim.2025.101970 40997623

[12] DANE. Estratificación socioeconómica para servicios públicos domiciliarios. 2025 [cited 2025 Sep 15]. Available from: https://www.dane.gov.co/index.php/servicios-al-ciudadano/servicios-informacion/estratificacion-socioeconomica.

[13] DANE. Censo Nacional de Población y Vivienda 2018. 2018 [cited 2025 Oct 30]. Available from: https://www.dane.gov.co/index.php/estadisticas-por-tema/demografia-y-poblacion/censo-nacional-de-poblacion-y-vivenda-2018.

[14] Alleva JM, Sheeran P, Webb TL, Martijn C, Miles E. A meta-analytic review of stand-alone interventions to improve body image. PLoS One. 2015;10(9):e0139177. 26418470 10.1371/journal.pone.0139177 PMC4587797

[15] Rubin AM. An examination of television viewing motivations. Commun Res. 1981;8(2):141–65.

[16] Mendelson BK, Mendelson MJ, White DR. Body-esteem scale for adolescents and adults. J Pers Assess. 2001;76(1):90–106. 11206302 10.1207/S15327752JPA7601_6

[17] Andres FE, Thornborrow T, Bowie WN, Coneo AMC, de la Rosa G, Evans EH, Fontalvo Acuña LS, Kolar DR, Mebarak M, Tovar Castro JC & Boothroyd LG. Validation of a Latin American Spanish version of the Body Esteem Scale for Adolescents and Adults (BESAA-LA) in Colombian and Nicaraguan adults. J Eat Disord. 2023;11(1):219. 10.1186/s40337-023-00942-5 PMC10709846 38066645

[18] Schaefer LM, Thompson JK. The development and validation of the Physical Appearance Comparison Scale–3 (PACS-3). Psychol Assess. 2018;30(10):1330–41. 29781660 10.1037/pas0000576 PMC6942695

[19] Schaefer LM, Burke NL, Thompson JK, Dedrick RF, Heinberg LJ, Calogero RM, et al. Development and validation of the sociocultural attitudes towards appearance questionnaire-4 (SATAQ-4). Psychol Assess. 2015;27(1):54. 25285718 10.1037/a0037917

[20] Villegas Moreno MJ, Londoño Pérez C, Pardo Adames C. Validation of the Sociocultural Attitudes Questionnaire on Appearance (SATAQ-4) in the Colombian population. Acta Colomb Psicol. 2021;24(1):86.

[21] Walker DC, Gaither SE, De Los Santos B, Keigan J, Schaefer LM, Thompson JK. Development and validation of a measure of curvy ideals internalization. Body Image. 2022;43:217–31. 36191379 10.1016/j.bodyim.2022.09.005 PMC9750804

[22] McCreary DR, Sasse DK, Saucier DM, Dorsch KD. Measuring the drive for muscularity: factorial validity of the drive for muscularity scale in men and women. Psychol Men Masc. 2004;5(1):49–58.

[23] Sepulveda AR, Parks M, de Pellegrin Y, Anastasiadou D, Blanco M. Validation of the Spanish version of the Drive for Muscularity Scale (DMS) among males: confirmatory factor analysis. Eat Behav. 2016;1(21):116–22. 10.1016/j.eatbeh.2016.01.010 26829369

[24] Gideon N, Hawkes N, Mond J, Saunders R, Tchanturia K, Serpell L. Development and psychometric validation of the EDE-QS, a 12 item short form of the Eating Disorder Examination Questionnaire (EDE-Q). PLoS One. 2016M;11(5):e0152744. 27138364 10.1371/journal.pone.0152744 PMC4854480

[25] Peláez-Fernández MA, Labrador FJ, Raich RM. Validation of eating disorder examination questionnaire (EDE-Q)–Spanish version–for screening eating disorders. Span J Psychol. 2012;15(2):817–24. 22774455 10.5209/rev_sjop.2012.v15.n2.38893

[26] Campo-Arias A, Miranda-Tapia GA, Cogollo Z, Herazo E. Reproducibilidad del Índice de Bienestar General (WHO-5 WBI) en adolescentes estudiantes. Rev Científica Salud Uninorte. 2015 Mar 24 [cited 2024 Nov 5];31(1). Available from: https://rcientificas.uninorte.edu.co/index.php/salud/article/view/5493.

[27] Blom EH, Bech P, Högberg G, Larsson JO, Serlachius E. Screening for depressed mood in an adolescent psychiatric context by brief self-assessment scales–testing psychometric validity of WHO-5 and BDI-6 indices by latent trait analyses. Health Qual Life Outcomes. 2012Dec 11;10(1):149. 23227908 10.1186/1477-7525-10-149 PMC3575311

[28] Campo-Arias A, Miranda-Tapia GA, Cogollo Z, Herazo E. Reproducibilidad del Índice de Bienestar General (WHO-5 WBI) en estudiantes adolescentes. Rev Salud Uninorte. 2015;31(1):18–24.

[29] Harvey RD, Tennial RE, Hudson BK. The development and validation of a colorism scale. J Black Psychol. 2017Oct 1;43(7):740–64.

[30] R Core Team. R: A language and environment for statistical computing.. R Foundation for Statistical Computing, Vienna, Austria; 2021 [cited 2024 Oct 31]. Available from: https://www.R-project.org/.

[31] Little RJA. A test of missing completely at random for multivariate data with missing values. J Am Stat Assoc. 1988Dec 1;83(404):1198–202.

[32] Woods AD, Gerasimova D, Van Dusen B, Nissen J, Bainter S, Uzdavines A, et al. Best practices for addressing missing data through multiple imputation. Infant Child Dev. 2024;33(1):e2407.

[33] Staudt A, Freyer-Adam J, Ittermann T, Meyer C, Bischof G, John U, et al. Sensitivity analyses for data missing at random versus missing not at random using latent growth modelling: a practical guide for randomised controlled trials. BMC Med Res Methodol. 2022Sep 24;22(1):250. 36153489 10.1186/s12874-022-01727-1 PMC9508724

[34] Cohen J. Statistical Power Analysis for the Behavioral Sciences. 2nd ed. New York: Routledge; 1988. p. 567.

[35] República de Colombia. SUIN Juriscol. 2024 [cited 2025 Oct 30]. Ley 2383 de 2024: Por medio de la cual se promueve la educación socioemocional de los niños, niñas y adolescentes en las instituciones educativas de preescolar, primaria, básica y media en Colombia. Available from: https://www.suin-juriscol.gov.co/.

