# Supplementary material for: Effects of a facilitator-delivered, group-based school intervention to improve media literacy and body dissatisfaction among adolescents: protocol of a cluster randomized controlled trial in Colombia
Source: Trials. 2026 Jan 22;27:138. doi: 10.1186/s13063-026-09460-6 (PMC12910830; doi:10.1186/s13063-026-09460-6)
Supplement: Supplementary file 1 — Additional file 1. Supplementary Materials S1-list of questionnaires [file 13063_2026_9460_MOESM1_ESM.docx]

# Supplementary Materials S2 – list of questionnaires

## Body Esteem Scale for Adolescents and Adults (BESAA)

English version: Mendelson et al., 2001

Spanish version: Andres et al., 2023

## Perceived Reality Scale – Social Media (PRS-SM)

English version: Rubin, 1981

Spanish version: Boothroyd et al., (under review)

## Curvy Ideal Internalization (CII)

English version: Walker et al., 2022

Spanish version: Andres et al., (under review)

## Eating Disorder Examination Questionnaire (EDE-Q)

English version: Luce & Crowther, 1999

Spanish version: Peláez-Fernández et al., 2012

## Multidimensional Comparison Attitudes towards Appearance (M-PACS)

English version: Manuscript in preparation

Spanish version: Manuscript in preparation

## Risky Appearance Altering Behaviours Inventory (RAABI)

English version: Andres et al., (under review)

Spanish version: Andres et al., (under review)

## Sociocultural Attitudes Towards Appearance Questionnaire (SATAQ-4)

Englihs version: Schaefer et al., 2015

Spanish version: Villegas Moreno et al., 2021

## Colourism scale (CS)

English version: Harvey et al., 2017

Spanish version: manuscript in preparation

## Drive for Muscularity (DMS)

English version: McCreary et al., 2004

Spanish version: Sepulveda et al., 2016

## WHO general well-being

English version: Blom et al., 2012

Spanish version: Campo-Arias et al., 2015

# References

Andres, F. E., Thornborrow, T., Bowie, W. N., Coneo, A. M. C., de la Rosa, G., Evans, E. H., Acuña, L. S. F., Kolar, D. R., Chams, M. R. M., Castro, J. C. T., & Boothroyd, L. G. (2023). Validation of a Latin American Spanish version of the Body Esteem Scale for Adolescents and Adults (BESAA-LA) in Colombian and Nicaraguan adults. *Journal of Eating Disorders*, *11*(1). https://doi.org/10.21203/rs.3.rs-3116832/v1

Blom, E. H., Bech, P., Högberg, G., Larsson, J. O., & Serlachius, E. (2012). Screening for depressed mood in an adolescent psychiatric context by brief self-assessment scales – testing psychometric validity of WHO-5 and BDI-6 indices by latent trait analyses. *Health and Quality of Life Outcomes*, *10*(1), 149. https://doi.org/10.1186/1477-7525-10-149

Campo-Arias, A., Miranda-Tapia, G. A., Cogollo, Z., & Herazo, E. (2015). Reproducibilidad del Índice de Bienestar General (WHO-5 WBI) en adolescentes estudiantes. *Revista Científica Salud Uninorte*, *31*(1), Article 1. https://doi.org/10.14482/sun.31.1.5493

Harvey, R. D., Tennial, R. E., & Hudson Banks, K. (2017). The Development and Validation of a Colorism Scale. *Journal of Black Psychology*, *43*(7), 740–764. https://doi.org/10.1177/0095798417690054

Luce, K. H., & Crowther, J. H. (1999). The reliability of the eating disorder examination—Self‐report questionnaire version (EDE‐Q). *International Journal of Eating Disorders*, *25*(3), 349–351. https://doi.org/10.1002/(SICI)1098-108X(199904)25:3<349::AID-EAT15>3.0.CO;2-M

McCreary, D. R., Sasse, D. K., Saucier, D. M., & Dorsch, K. D. (2004). Measuring the Drive for Muscularity: Factorial Validity of the Drive for Muscularity Scale in Men and Women. *Psychology of Men & Masculinity*, *5*(1), 49–58. https://doi.org/10.1037/1524-9220.5.1.49

Mendelson, B. K., Mendelson, M. J., & White, D. R. (2001). Body-esteem scale for adolescents and adults. *Journal of Personality Assessent*, *76*(1), 90–106. https://doi.org/10.1207/S15327752JPA7601_6

Peláez-Fernández, M. A., Labrador, F. J., & Raich, R. M. (2012). Validation of eating disorder examination questionnaire (EDE-Q)–Spanish version–for screening eating disorders. *The Spanish Journal of Psychology*, *15*(2), 817–824. https://doi.org/10.5209/rev_SJOP.2012.v15.n2.38893

Rubin, A. M. (1981). An Examination of Television Viewing Motivations. *Communication Research*, *8*(2), 141–165. https://doi.org/10.1177/009365028100800201

Schaefer, L. M., Burke, N. L., Thompson, J. K., Dedrick, R. F., Heinberg, L. J., Calogero, R. M., Bardone-Cone, A. M., Higgins, M. K., Frederick, D. A., & Kelly, M. (2015). Development and validation of the sociocultural attitudes towards appearance questionnaire-4 (SATAQ-4). *Psychological Assessment*, *27*(1), 54. https://doi.org/10.1037/a0037917

Sepulveda, A. R., Parks, M., de Pellegrin, Y., Anastasiadou, D., & Blanco, M. (2016). Validation of the Spanish version of the Drive for Muscularity Scale (DMS) among males: Confirmatory factor analysis. *Eating Behaviors*, *21*, 116–122. https://doi.org/10.1016/j.eatbeh.2016.01.010

Villegas Moreno, M. J., Londoño Pérez, C., & Pardo Adames, C. (2021). Validation of the Sociocultural Attitudes Questionnaire on Appearance (SATAQ-4) in the Colombian population. *Acta Colombiana de Psicología*, *24*(1), 86. https://doi.org/10.14718/acp.2021.24.1.8

Walker, D. C., Gaither, S. E., De Los Santos, B., Keigan, J., Schaefer, L. M., & Thompson, J. K. (2022). Development and validation of a measure of curvy ideals internalization. *Body Image*, *43*, 217–231. https://doi.org/10.1016/j.bodyim.2022.09.005
